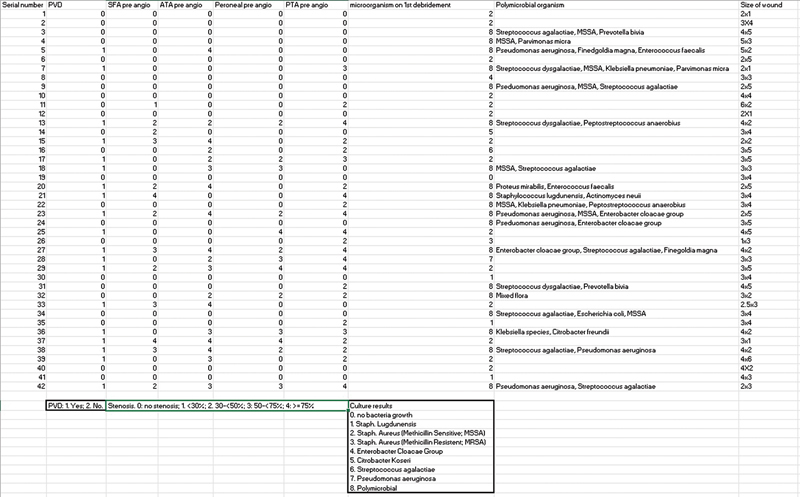# Outcome of Rotation Flap Combined with Incisional Negative Pressure Wound Therapy for Plantar Diabetic Foot Ulcers

**DOI:** 10.1055/a-2544-2938

**Published:** 2025-05-15

**Authors:** Jiajun Feng, Coeway Boulder Thng, Jason Wong, Quah Mei Fern Alison, Nicole Tao Ying Lim, Francis Keng Lin Wong, Kimberley Leow, Leon Timothy Charles Alvis, Sum Leong, Farah Gillan Irani, Wenxian Png, Eric Wei Liang Cher, Yee Onn Kok, Allen Wei-Jiat Wong, Khong Yik Chew

**Affiliations:** 1 Department of Plastic, Reconstructive, and Aesthetic Surgery, Sengkang General Hospital, Singapore, Singapore; 2 Department of Plastic Surgery and Burns, University Hospital of South Manchester, England; 3 Department of Orthopaedic Surgery, Sengkang General Hospital, Singapore, Singapore; 4 Department of Podiatry, Sengkang General Hospital, Singapore, Singapore; 5 Department of Vascular and Interventional Radiology, Sengkang General Hospital, Singapore, Singapore

**Keywords:** diabetic ulcer, diabetic limb salvage, local flap, incisional NPWT, plantar

## Abstract

**Background** Diabetic foot ulcers (DFUs) affect approximately 20% of diabetic patients and pose significant risks, especially for plantar wounds that bear weight. Conventional treatments often have suboptimal results, necessitating the exploration of reconstructive options. Plastic surgery interventions, such as skin grafts and flaps, have shown promising outcomes, but with considerable complications. This study evaluates the efficacy of rotation flap reconstruction with incisional negative pressure wound therapy (NPWT) for plantar DFUs.

**Methods** We conducted a retrospective review of 42 patients who underwent rotation flap closure for plantar DFUs. We optimized the preoperative conditions with aggressive infection control and vascular assessment. We performed rotation flaps with incisional NPWT as the operative technique. We managed the postoperative conditions with offloading continuous incisional NPWT and footwear.

**Results** All patients achieved initial wound healing, with a median duration of 36 days. Complications occurred in 14% of cases. The recurrence rate was 21% during follow-up, which was significantly higher in patients with Charcot foot deformity. We present three illustrative cases that demonstrate the efficacy of rotation flaps.

**Conclusion** Rotation flap closure, supplemented by incisional NPWT, emerges as a viable option for plantar DFUs, achieving high initial healing rates, low complications, and reduced recurrence. Notably, patients with Charcot foot deformity require more attention and intervention to prevent recurrence.

## Introduction

Diabetic foot ulcers (DFUs) pose a common and significant risk to diabetic patients, affecting approximately 20% of them throughout their lifetimes. 1 These ulcers are the result of a combination of factors, including neuropathy, peripheral arterial disease (PAD), deformities, infections, and impaired wound healing. 2 Among DFUs, plantar wounds are particularly challenging due to their weight-bearing demands and tendency to recur. 3

Current recommended treatments, primarily conservative dressing, 4 often yield suboptimal results, high dressing utility, slow healing rates, prolonged treatment durations, high recurrence rates, local wound infection, and complications leading to amputations. 5 The occurrence of a major amputation in DFU increases the 5-year mortality from 30.5 to 56.6%. 6 Robust healing of plantar ulcers is therefore of paramount importance. The option to reconstruct these complex wounds and potentially prevent major amputations has profound effects on the prognosis of a patient. Plastic surgery interventions, such as skin grafts, 7 local flaps, 8 and free flaps, 9 can positively impact these outcomes. However, skin grafts are less reliable for plantar wounds, and pedicled and free flaps are complex options typically reserved for larger defects. Abdelfattah et al popularized the use of thin “expendable” free soft tissue reconstructions like the superficial circumflex iliac artery perforator flap to improve outcomes, 10 yet the complexity of free flaps remains a significant limitation.

A few publications have explored the use of local flaps for plantar wound coverage, albeit with common issues, including prolonged nonweight-bearing periods leading to noncompliance, wound dehiscence due to high tension, and infection, resulting in complication rates ranging from 40 to 60%. 8 11 12 In our multidisciplinary limb salvage service, we conducted a retrospective review of plantar wound coverage using rotation flaps enhanced by incisional negative pressure wound therapy (NPWT) to analyze outcomes and establish this technique as an optimal choice for selected diabetic patients with plantar wounds.

## Methods

### Patient Selection and Management

Institutional Review Board (IRB ref: 2023/2248) exemption was approved by the IRB board in Sengkang General Hospital for clinical audit. Informed consent was obtained from patients to publish their photographs.

**Fig. 1:**
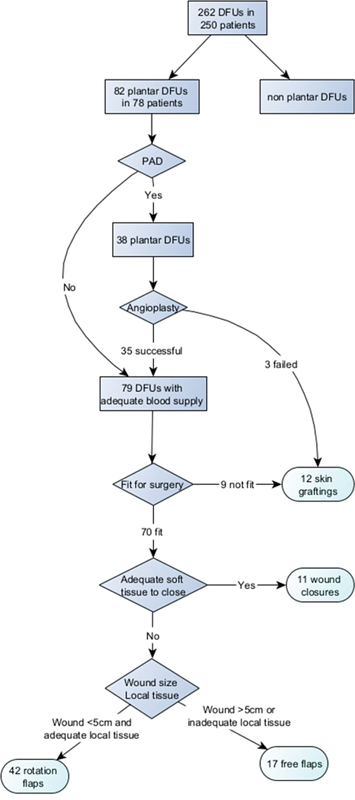
Forty-two plantar diabetic foot ulcers (DFUs) received rotation flap coverage out of 262 DFUs. The indications for rotation flap include wound size < 5 cm with adequate local soft tissue, and adequate blood supply without peripheral arterial disease (PAD) or with PAD after successful angioplasty.

We conducted a retrospective review of all patients treated by the Diabetic Limb Salvage Service in Sengkang General Hospital, which offers comprehensive wound-focused multidisciplinary care for DFU patients. Demographics including age and gender, comorbidities including peripheral vascular disease, renal disease, heart disease, HbA1c in diabetes mellitus, and DFU characteristics including WIfI (Wound, Ischemia, foot Infection) score, staging, and presence of Charcot foot deformity, were collected. Between March 2020 and July 2023, we treated 250 patients with a total of 262 DFUs. Each patient underwent Peripheral Arterial Duplex Assessment to evaluate their lower limb vascular status, as well as wound debridement(s). For patients with significant PAD, significant being defined as stenosis >50%), angioplasty was performed to enhance blood flow. Following debridement and infection control, and based on the results of angioplasty, a coverage plan was developed. For patients without PAD or with PAD that underwent successful angioplasty, primary closure was undertaken for patients with adequate soft tissue around the wound. For DFUs smaller than 5 cm and with sufficient local tissue, local flaps were utilized (see Supplementary Data 1 , available in online version only). In cases where the wound was larger than 5 cm or where there was insufficient local tissue, free flaps were employed. Patients who did not achieve satisfactory results from angioplasty were managed conservatively using NPWT, with eventual split-thickness skin grafting as necessary. In short, the selection criteria of plantar DFU for rotation flap reconstruction is wound size <5 cm with adequate local soft tissue and adequate blood supply without PAD or with PAD but after successful angioplasty ( Fig. 1 ). Among these, 78 patients had 82 plantar wounds, and 42 of them underwent rotation flap closure. Additionally, 11 patients had direct wound closure, 12 received skin grafts, and 17 underwent free flap reconstruction. The 42 patients who received rotation flap coverage were included in this study with an average follow-up duration of 11.5 months (ranging from 3 to 30 months). Outcome measures including wound healing rate and duration, wound recurrence, and amputation were collected.

### Preoperative Optimization

All patients underwent wound debridement and received NPWT with instillation and dwell (NPWT-id; Veraflo [KCI USA, Inc., San Antonio, TX]). Dwell time: 10 minutes, NPWT time: 3.5 hours at −125 mm Hg, solution: Granudacyn (Molnlycke Sweden, Inc., Gothenburg) to expedite local infection control. Empirical antibiotics were initiated, followed by targeted therapy for positive tissue cultures (see Supplementary Data 1 , available in online version only). Patients who presented with osteomyelitis with positive bone cultures were given a prolonged course of antibiotics for 6 weeks. Wound coverage was only performed after the inflammatory marker (C-reactive protein) normalized and the wound granulation appeared, indicating infection resolution, with a mean duration of 8.8 (3.1, range: 4–15) days from initial debridement. In cases of Charcot foot disease, orthopaedic surgeons performed exostectomies during wound debridement for exposed bony prominence. All patients received vascular assessment via arterial duplex scans. Patients with significant arterial stenosis (>50% in femoral, anterior tibial, posterior tibial, and peroneal arteries) underwent angioplasties to augment arterial inflow before wound reconstruction. Successful angioplasties were defined as <30% stenosis with good perfusion postangioplasty.

### Operative Techniques

**Fig. 2:**
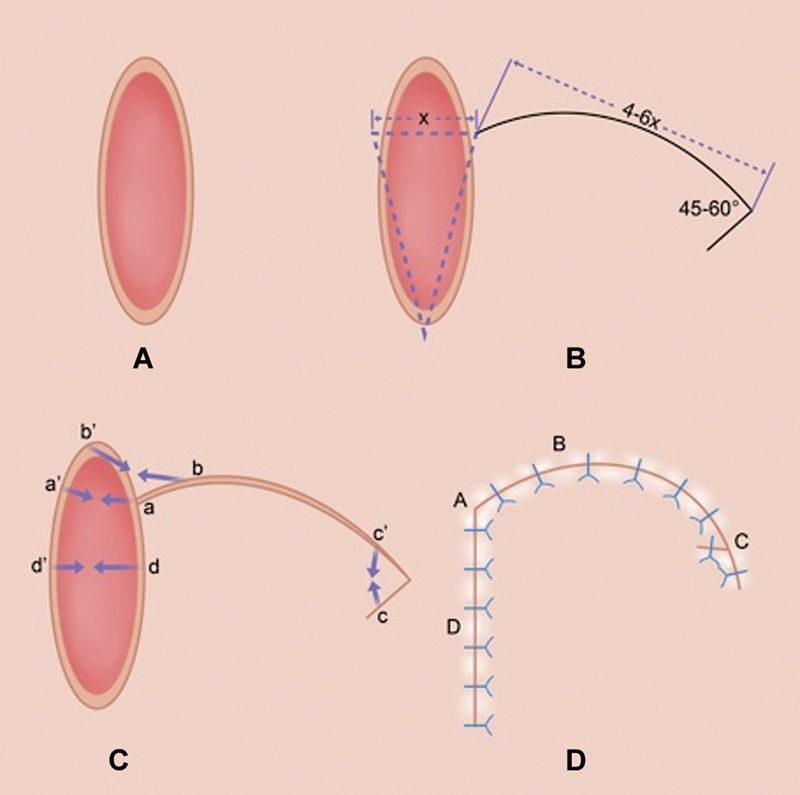
Rotation flap design for diabetic foot ulcer (DFU) plantar wounds. ( **A** ) An elliptical plantar wound. ( **B** ) Intralesional triangulation (dotted line) for rotation flap design (solid line). With a defect size of X, the flap size will be 4–6X. ( **C** ) Rotation flap with VY closure with angle between 45 and 60 degrees (flap edge: a, b, and d. Wound edge: a', b', c, c', and d'. Arrows indicating directions of tissue movement for closure). ( **D** ) Final closure after local flap reconstruction (approximated closure points: a and a' to A; b and b' to B; c and c' to C; d and d' to D).

A rotation flap was designed, beginning with intradefect triangulation ( Fig. 2 ). The size of the flap is designed to be 4 to 6 times the size of the defect. The flap was raised either laterally or medially based on tissue availability and to avoid weight-bearing areas, ending with a burrow triangle or VY advancement with angles between 45 and 60 depending on tissue laxity to facilitate donor-site closure while advancing the flap. The flap was undermined at the subfascial plane by releasing retaining ligaments, and the surrounding tissues were undermined at the subcutaneous plane to enable closure with acceptable tension. Minimal internal sutures (2–3) were used to approximate the key points during flap inset. A Blake (Johnson & Johnson USA, Inc., New Brunswick, NJ) drain was always placed to drain the undermined space. Direct closure with unabsorbable sutures and staples was performed. Incisional NPWT was applied to reduce wound tension and facilitate tissue recruitment toward the suture line. Hypafix tape was often used to augment tissue recruitment over the NPWT dressing and further reduce tension on the suture line.

### Postoperative Management

Patients were instructed to avoid weight-bearing on the operated leg for the first 7 days. Wound cultures were monitored, and antibiotics were adjusted accordingly. After the first wound inspection on postoperative day 7 (POD7) to ensure satisfactory union, patients began ambulation, wearing an aircast boot with PegAssist Insole (DARCO USA, Inc., Sunrise, FL), which offloaded the plantar suture lines, in addition to the incisional NPWT (−125 mm Hg), continued for another 21 days to ensure adequate wound healing due to the limited healing capacity of the DFUs. Once patients were assessed by physiotherapy and deemed suitable for home care, they were discharged. Patients returned weekly for incisional NPWT change and for suture removal on POD21. After finishing a total of 28 days of NPWT dressing, patients were advised to continue using the aircast boot until a podiatrist reviews to provide offloading footwear. While wearing the recommended footwear, F-scan (Tekscan USA, Inc., Norwood, MA) was performed and if there were any areas of high pressure, a total contact insole or orthosis was considered. 13 The antibiotic treatment was continued for 2 weeks after wound closure or for 6 weeks, if there were positive bone cultures.

A healed wound is defined as the full epithelialization of wound and wound recurrence is defined as a wound that reappears at the same site after having previously healed. Major lower limb amputation is defined as lower limb amputation above the ankle, such as the below-knee amputation, whereas minor amputation is defined as amputation below the ankle. The WIfI system 14 was used to assess the wounds at initial presentation. PAD is defined as greater than 50% stenosis in any of the affected lower limb arteries on arterial duplex scan.

### Statistical Analysis

All statistical analyses were conducted using IBM SPSS Statistics ver. 20.0 software (IBM Co., Armonk, NY). Continuous variables were presented as median (± median absolute deviation) or mean (± standard deviation) as appropriate, whereas categorical variables were presented as proportions. The Modified Moody's median test was utilized for median value comparisons, the Z test was employed for proportional tests, and the Student's *t* -test was used for mean variables. A significance value of *p* < 0.05 was considered statistically significant.

## Results

### Demographics and Comorbidities

Among the 42 patients who underwent rotation flap reconstruction for their plantar wounds, the mean age was 60 (36–79) years, with 50% being male. Of the limbs, 45% (19/42) had PAD (see Supplementary Data 1 , available in online version only), and all of them underwent successful angioplasties. The mean HbA1c was 8.8% (normal range: 4–5.6%), and 5% (2/42) had end-stage renal failure (ESRF), whereas 36% (15/42) had chronic kidney disease. Additionally, 33% (14/42) had ischemic heart disease, and 33% (14/42) had Charcot foot disease. For the wounds, 41 patients had exposed critical structures including bone, joint, or tendon (WIfI wound score 2) and 1 patient had calcaneal involvement (WIfI wound score 3). There were 19 forefoot, 17 midfoot, and 6 heel wounds. At presentation, 48% (20/42) had WIfI grade 4 DFUs, 43% (18/42) had grade 3, and 10% (4/42) had grade 2 ( Table 1 ).

**Table 1:** Demographic and comorbidities of patients' population

Characteristics ( *n* = 42)	Value
Gender (male)	21 (50%)
Age (mean ± SD)	60 ± 10
HbA1c (mean ± SD)	8.8 ± 2.75
Peripheral artery disease and angioplasty	19/42 (45%)
ESRF	2/42 (5%)
CKD	15 (36%)
IHD	14 (33%)
Charcot foot deformity	14 (33%)
WIfI stage	
2	4 (10%)
3	18 (43%)
4	20 (48%)

Abbreviations: CKD, chronic kidney disease; ESRF, end-stage renal failure; IHD, ischemic heart disease; SD, standard deviation; WIfI, Wound, Ischemia, foot Infection.

### Outcomes

All 42 plantar wounds reconstructed using rotation flaps healed initially during the 11.5-month (3–30) follow-up period, with a median wound healing duration of 36 (24) median (SD) (range: 14–129) days. Out of these, 14% (6/42) patients experienced complications related to the flap surgery, with two cases of infection requiring further debridement and secondary closure, and four cases of partial wound dehiscence due to tension that subsequently healed with dressings and offloading. After the initial wound healing, 21% (9/42) of patients developed a recurrence of their plantar DFUs. Five of them healed (three after dressing and offloading, and the other two with debridement followed by secondary closure). However, four of the nine patients with recurrences remained to have active wounds at the end of the follow-up period. Two of them were managed conservatively with dressings, whereas the other two suffered infections requiring further surgical interventions (one ray amputation and one free flap coverage). No major amputations were performed to treat the recurrent DFUs ( Table 2 ).

**Table 2:** Outcome of rotation flap combined with incisional negative pressure wound therapy on plantar diabetic foot ulcers

Outcome ( *n* = 42)	Value
Rate of wound healing	42/42 (100%)
Duration of wound healing (median ± standard deviation)	36 ± 24 d
Complications	6 (14%)
Recurrence	9 (21%)
Active wound on follow-up	4 (10%)
Major amputation	0 (0%)
Minor amputation	1 (2.4%)

In subgroup analysis, patients with Charcot foot deformity (14/42) had significantly higher rates of wound recurrence (43%; 6/14) and nonhealing wounds (29%; 4/14), compared with non-Charcot foot group (11% [3/28] and 0% [0/28]; *p* = 0.017 and 0.003, respectively; Table 3 ).

**Table 3:** Outcome comparison between patients with Charcot foot deformity versus without Charcot foot deformity

	Non-Charcot foot ( *n* = 28)	Charcot foot ( *n* = 14)	*p* -Value
Wound healing duration	39.8 ± 22	45.3 ± 27	0.48
Recurrence	3 (11%)	6 (43%)	0.017
Active wound	0 (0%)	4/14 (29%)	0.003

For the 31 patients with follow-up HbA1c levels available between 3- and 6-month postsurgery, a significant improvement was observed, with HbA1c levels decreasing from 9.1% (2.6) to 7.2% (1.8) ( *p* = 0.000496).

### Additional Procedures

Additional orthopaedic procedures were recorded such as tendoachilles lengthening ( *n* = 4), and corrective orthopaedic interventions ( *n* = 2). Tarsal tunnel release procedures were performed in patients with symptomatic neuropathy and positive Tinel's sign ( *n* = 8), either simultaneously with wound closure or posthealing.

**Fig. 3:**
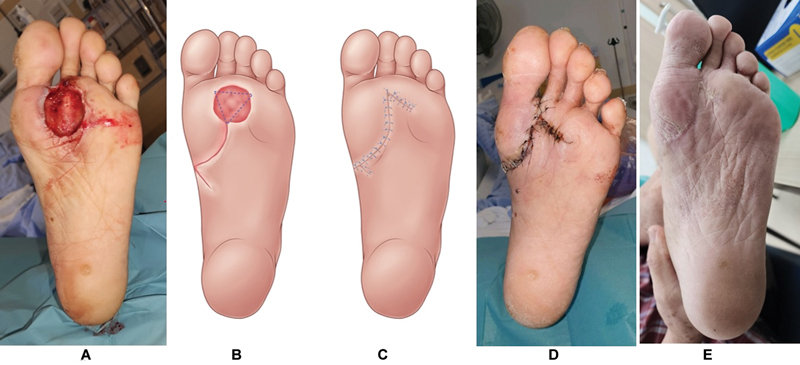
Patient A with forefoot wound reconstructed with rotation flap. ( **A** ) A 3 cm × 4 cm defect exposing second metatarsophalangeal joint. ( **B** ) Rotation flap design over lax tissue at medial instep. ( **C** ) Flap inset. ( **D** ) One week postoperatively before starting ambulation. ( **E** ) One month postoperatively with complete wound healing while ambulating.

Three case studies illustrate the use of rotation flaps in diabetic plantar wound reconstruction:

Case 1 had a deep forefoot plantar ulcer with systemic inflammatory response syndrome (SIRS) and a WIfI grade 4 DFU. The resultant defect postdebridement measured 3 cm × 4 cm with exposed metatarsophalangeal joints. A rotation flap was used to reconstruct the plantar ulcer, which subsequently healed without complications ( Fig. 3 ).

**Fig. 4:**
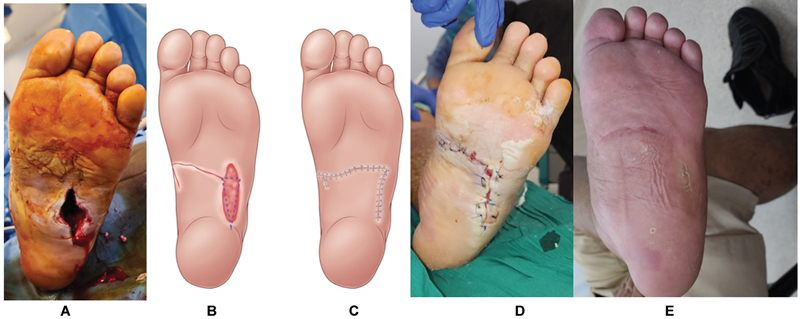
Patient B with Charcot foot deformity and a plantar ulcer measuring 2 cm × 5 cm with exostectomy performed during debridement. ( **A** ) The midfoot defect with exposed bone at wound base. ( **B** ) Rotation flap design over medial instep. ( **C** ) Flap inset to reconstruct the defect. ( **D** ) One week postoperatively, wound was healing well before starting ambulation. ( **E** ) Healed wound at one month while ambulating, wearing prescribed off-loading footwear (seen in background).

Case 2 presented with an extensive midfoot plantar abscess and SIRS with a background of Charcot foot (WIfI grade 4). The defect postdebridement measured 3 cm × 5 cm with exposed bone and joints. A rotation flap was used for wound reconstruction, leading to an uneventful healing process, enabling the resumption of ambulation with a total contact insole ( Fig. 4 ).

**Fig. 5:**
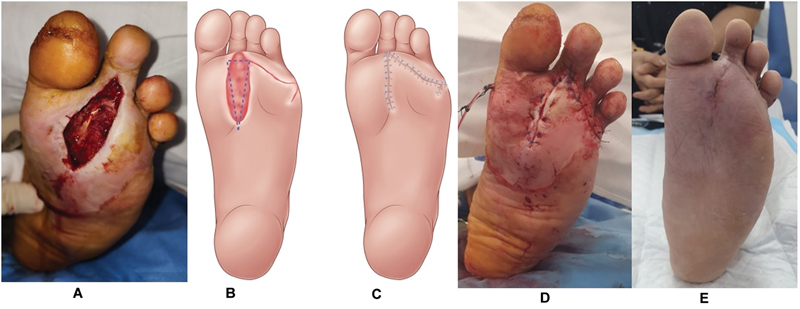
Patient C with end-stage renal failure and previous 5th ray amputation presented with ( **A** ) a 5 cm × 3 cm forefoot wound exposing tendon and bone. ( **B** ) Rotation flap over lateral forefoot laxity from previous ray amputation. ( **C** ) Flap inset to reconstruct the defect. ( **D** ) Wound closure intraoperatively. ( **E** ) Wound well healed 6 weeks postoperatively while ambulating on customized footwear.

Case 3 had extensive forefoot infection and SIRS (WIfI grade 4 DFU) with past medical history including a previous fifth toe ray amputation and ESRF. The resultant forefoot wound measured 2 cm × 5 cm. It was covered with a rotation flap, leading to complete healing, and the patient regained her premorbid ambulatory status ( Fig. 5 ).

## Discussion

Our study demonstrates the safe and efficient reconstruction of plantar wounds using rotation flap. We achieved a remarkable 100% initial wound healing rate after the flap reconstruction with a median duration of 36 days, surpassing the literature's reported median plantar DFU wound closure rate of 77% at 3 months 15 with conventional treatment. In addition to the impressive healing rate, our approach also resulted in a lower recurrence rate of 21% compared with the 30 to 40% recurrence rates reported in the literature, 3 5 15 which, in turn, contributed to reduced major and minor amputations.

These favorable outcomes can be attributed to several key advantages of rotation flap reconstruction. First, this method utilizes tissue reconstruction that is like-for-like, providing the necessary durability for ambulation and the internal structural support required during ambulation. 16 Second, the utilization of rotation flaps simplifies the surgical procedure, eliminating an extra donor wound in skin grafting and the complexity associated with free flap coverage. Finally, our protocol incorporates incisional NPWT 17 18 and offloading, allowing for early and safe ambulation as early as POD7, in contrast to the commonly recommended wait of 21 days found in the literature. 11 19 This early ambulation significantly contributes to improved patient compliance and rehabilitation, resulting in a 100% patient compliance rate, and regaining ambulatory status, while maintaining excellent wound healing outcomes. Furthermore, 90% (38/42) of patients remained ambulatory without wound at the end of this study, whereas the remaining 10% (4/42) ambulated with assistance to offload the active plantar ulcer.

However, the application of local flaps for primary wound closure has traditionally been limited due to a high complication rate, varying between 40 and 60% as reported in various literature sources. 11 12 This elevated complication rate can be attributed to factors such as poor wound healing potential, infection, vascular insufficiency, tension, and wound dehiscence. Our treatment approach effectively addresses these factors, leading to improved wound closure rates and a reduction in complications.

Hyperglycemia has been found to be detrimental to wound healing. 20 Our study places a strong emphasis on strict diabetic control throughout the treatment process with twice weekly medication adjustment augmented with dietary modification by a dietitian. Wound closure was only considered when patients achieved acceptable glucose control. Furthermore, all patients with HbA1c levels exceeding 8.0% received an outpatient dietitian review and were provided with self-glucose monitoring devices. This proactive approach led to a significant reduction in HbA1c levels within our patient cohort from 9.1 to 7.2%, contributing to the high rate of wound healing.

For infected DFUs, achieving infection-free wounds is a prerequisite for flap surgery. Our protocol was meticulously designed to systematically address this issue. All patients were initiated on empirical antibiotics and underwent wound debridement within 24 hours to minimize the impact of infection. NPWT with instillation system of Granudacyn solution was applied either intraoperatively or on POD1 to treat the infection. 21 Culture-directed antibiotics were adjusted as soon as debridement culture results became available. The continuous treatment with antibiotics and topical antiseptics effectively eliminated residual infection, leading to a transition from catabolism to anabolism, marked by normalizing C-reactive protein levels. 22 Such a metabolic transition was critical for improving angiogenesis 23 and wound healing potential, 24 which were essential for successful DFU reconstruction.

Intraoperatively, to minimize foreign material as a nidus for infection, we employed minimal internal sutures with antibiotic-coated absorbable sutures (Vicryl 2/0; Johnson & Johnson USA, Inc., New Brunswick, NJ), and a Blake drain was always placed to ensure the drainage of hematoma and seroma. Adequate duration of antibiotic treatment (2 or 6 weeks) was given to ensure the complete resolution of infection and optimal wound healing. With these protocols in place, we achieved a lower infection rate of 5% (2/42) after rotation flap reconstruction.

The success of flap procedures is critically dependent on addressing vascular insufficiency. PAD is common in DFUs, affecting up to 40 to 50% of cases. In our series, 45% (19/42) of patients had peripheral artery disease, and all of them received successful angioplasty within 7 days before wound closure to ensure excellent arterial inflow with minimal restenosis. The flap design maintained sufficient dermal and subdermal plexus blood supply to the flap. Limited undermining helped preserve perforators, and the inclusion of the medial plantar perforator at the flap base was preferred during the design. In addition to good arterial inflow, venous outflow is equally important for flap survival. The preservation of a skin bridge and limited undermining helped maintain venous return. 25 With these considerations, the random flap maintained ample vascular supply, allowing for healing even when closed with some degree of tension.

Excessive tension at the wound edge is the primary cause of wound dehiscence. To minimize such complications, we initiated a multifaceted approach beginning with patient selection. Only patients with wounds width less than 5 cm and who had ample plantar soft tissue were chosen for rotation flap reconstruction. During surgery, incisions were strategically placed away from weight-bearing areas to minimize tension during future ambulation. In addition to flap undermining, we also undermined the surrounding wound edge to allow for mobilization of the surrounding soft tissue, thus reducing the tension required for closure. Internal sutures were employed to bring the wound edges together at the deep fascia level to reduce tension on skin closure.

Further tension relief was achieved by employing incisional NPWT following wound closure. Numerous studies have demonstrated the effectiveness of incisional NPWT in enhancing wound healing for high-tension closures, such as those in breast surgeries, 26 lower limb procedures, 27 abdominal, 28 and perineal wounds. 29 The dressing redistributes tension from the suture line to adjacent intact skin, promoting minimal tension at the suture line for healing. After the initial wound inspection on POD7, patients transitioned to weight-bearing on the operated site using an aircast boot. The boot not only immobilized and offloaded the plantar surface but also allowed incorporation of the incisional NPWT for an additional 3 weeks. Controlled weight-bearing enabled patients to be discharged home and to continue essential activities of daily living, thereby reducing noncompliance rates associated with nonweight-bearing protocols.

Through the combined implementation of these techniques, all 42 plantar wounds in our study successfully healed following rotation flap closure, with a complication rate of only 14% (6/42), which is notably lower than the 35 to 60% reported in existing literature. 11 12 19

### Recurrence

The management of DFUs has historically been plagued by a high annual recurrence rate of 40%. 3 5 Other studies have reported lower recurrence rates by using rotation flap for plantar ulcer coverage, but these studies only included patients with noninfected chronic DFUs. 11 19 Our study, however, included patients presenting with acute infections, in whom a higher baseline recurrence rate of 30 to 40% was anticipated, aligning with that of the broader DFU demographic.

To mitigate the recurrence of DFUs in our patient cohort, comprehensive podiatric assessments were conducted to ensure the provision of appropriate footwear. Despite these efforts, a recurrence rate of 21% (9/42) was observed. Initial recurrence management strategies included enhanced offloading techniques, such as the use of total contact insoles, orthoses, and personal mobility aids. These interventions lead to wound healing in five patients, whereas the remaining four, all of whom had Charcot foot deformity, continued to have persistent plantar wounds.

In subgroup analysis, it was observed that patients with Charcot foot deformity exhibited significantly higher recurrence rates and a higher rate of active wounds at follow-up ( Table 3 ). This finding aligns with that seen in the literature, 30 highlighting the high risk of DFUs with Charcot foot deformity and underscoring the importance of optimizing offloading and the need for corrective orthopaedic procedures.

### Limitations

This study is a single-center, retrospective review of rotation flap reconstruction. The cohort is relatively small, with a relatively short duration of follow-up. We plan to include more patients with longer follow-up in future studies.

### Conclusion

In conclusion, our study demonstrates that rotation flap closure is a safe and effective option for selected infected DFU patients. With our comprehensive protocol, early ambulation, high rates of wound healing, and low complication rates, are achievable. During follow-up, with proper offloading, low recurrence rates are observed in patients without Charcot foot deformity. On the other hand, the relatively high rate of recurrence and active wounds in patients with Charcot foot deformity underscores the importance of corrective orthopaedic reconstructive procedures to address the underlying structural deformities.

**Supplementary Data S1:**